# Insights and concerns on proteomic-based Mendelian randomization in ischemic stroke: the need for comprehensive data and methodological rigor

**DOI:** 10.1097/MS9.0000000000002812

**Published:** 2025-01-07

**Authors:** Xingcheng Zhu, Rongyuan Liu, Yuanzhi Fu, Dongqing Li, Jiarui Yang, Bo Chen, Jieming Zuo, Junhao Chen, Xiangyun Li

**Affiliations:** a Department of Clinical Laboratory, The Second People’s Hospital of Qujing City, Qujing, Yunnan, China; b Department of Obstetrics and Gynecology, Qujing Women’s and Children’s Hospital, Qujing, Yunnan, China; c Kunming University of Science and Technology, Kunming, Yunnan, China; d Department of Dermatology, Qujing Hospital, Kunming Medical University, Qujing, Yunnan, China; e Department of Hepatobiliary Surgery, The Second Affiliated Hospital of Kunming Medical University, Yunnan, China; f Department of Urology, The Second Affiliated Hospital of Kunming Medical University, Yunnan, China

*Dear Editor,*

We have carefully read the article by Zou *et al*^[1]^ published in the *International Journal of Surgery*. The authors employed Mendelian Randomization (MR) to evaluate drug targets related to proteomics and ischemic stroke (IS). Their primary findings suggest a causal relationship between plasma matrix metalloproteinase 12, SWAP70 protein, and cerebrospinal fluid prekallikrein protein with IS risk, which could inform early prevention and treatment strategies. This discovery enhances our understanding of the role of proteomics in IS pathogenesis and prevention. We commend the authors for their outstanding work and innovative approach. However, we have a few concerns and suggestions that we would like to share.

In Zou *et al*’s study, although the authors skillfully utilized multiple proteomics databases, including plasma protein pQTL and cerebrospinal fluid pQTL, and conducted a multicenter study, which undoubtedly strengthens the comprehensiveness and reliability of their analysis of the relationship between proteins and disease, there are still aspects of the study that warrant further discussion. Specifically, there appears to be a sample overlap between the plasma protein data (including 4775 proteins from the Fenland study, center 3) and the outcome data. The outcome data also used the Fenland study as a validation source, which may lead to false-positive results. Sample overlap violates one of the fundamental principles of MR, which requires the independence of exposure and outcome data to avoid confounding bias. Overlap can exaggerate the association between exposure and outcome, thereby undermining the validity of causal inference in MR analysis. Therefore, this issue needs careful consideration in the study design.

Second, the authors identified potential protein targets that exhibit a causal relationship with ischemic stroke. However, it remains to be explored whether these protein targets act independently, or if the effects of certain proteins are merely driven by their association with other proteins. In this context, I suggest that the authors incorporate Multivariable Mendelian Randomization (MVMR) in future research, where multiple related proteins are included as exposure variables simultaneously. This approach would allow for the adjustment of potential correlations between these exposure variables, thereby enabling the assessment of each protein’s independent contribution to the outcome. This is particularly important for understanding the multifaceted biological pathways involved in complex diseases, as many proteins may interact with one another, and analyzing each protein in isolation may overlook these interactions. MVMR can help reveal the independent effects of these protein targets, offering more accurate insights into potential therapeutic targets^[2,3]^. By implementing MVMR, the authors would not only validate the protein targets identified in univariable MR but also further distinguish those with independent contributions to ischemic stroke risk, providing a stronger foundation for targeted interventions.

Thirdly, the quality of the database used is crucial, and not merely the quantity. The recently published UK Biobank by Nature offers a more comprehensive and extensive data foundation, encompassing genetic associations with over 14,000 protein expression levels, 81% of which are novel discoveries^[4]^. This database’s scale and comprehensiveness far exceed the combined total of the two databases chosen by Zou et al. Future studies should consider incorporating the UK Biobank to obtain a more exhaustive proteomics database. However, the sheer size of this database may require advanced servers rather than standard computers for data processing, which could be one of the reasons why the authors did not use it.

Finally, while the authors significantly enhanced the reliability of their data by using outcome data from different sources as validation groups and incorporating meta-analysis, which provides a more solid foundation for their findings, it is worth noting that integrating multi-source data, though theoretically robust, may still suffer from sample overlap issues in practice. As mentioned in the second point, sample overlap could affect the accuracy of causal inference and introduce bias. To further strengthen the robustness of MR analysis, I suggest that in addition to Bayesian colocalization analysis, the authors consider introducing SMR colocalization analysis and conducting the HEIDI TEST^[5]^. These methods can effectively reduce potential bias caused by sample overlap, thereby enhancing the reliability and accuracy of causal inference. Moreover, intersecting the outcomes of Bayesian colocalization analysis with those from SMR colocalization analysis could further filter out more convincing protein targets, thus bolstering the robustness and credibility of the study’s results.

Overall, we appreciate the efforts and contributions made by Zou *et al* in this research. However, we have highlighted some concerns and areas for improvement that could help enhance future studies and provide more profound insights into targeted therapies.

## Data Availability

None.
